# Recombinant Adeno-Associated Virus-mediated rescue of function in a mouse model of Dopamine Transporter Deficiency Syndrome

**DOI:** 10.1038/srep46280

**Published:** 2017-04-18

**Authors:** P. Illiano, C.E. Bass, L. Fichera, L. Mus, E.A. Budygin, T.D. Sotnikova, D. Leo, S. Espinoza, R.R. Gainetdinov

**Affiliations:** 1 Department of Neuroscience and Brain Technologies, Fondazione Istituto Italiano di Tecnologia, 16163 Genova, Italy; 2 Department of Pharmacology and Toxicology, Jacobs School of Medicine and Biomedical Sciences, University at Buffalo, Buffalo, NY, USA; 3 Department of Advanced Robotics, Fondazione Istituto Italiano di Tecnologia, 16163 Genova, Italy; 4 Institute of Translational Biomedicine, St. Petersburg State University, St. Petersburg, 199034, Russia; 5 Department of Neurobiology and Anatomy, Wake Forest School of Medicine, Winston- Salem, NC, USA; 6 Skolkovo Institute of Science and Technology (Skoltech) Skolkovo, Moscow region, 143025, Russia

## Abstract

Dopamine Transporter Deficiency Syndrome (DTDS) is a rare autosomal recessive disorder caused by loss-of-function mutations in dopamine transporter (DAT) gene, leading to severe neurological disabilities in children and adults. DAT-Knockout (DAT-KO) mouse is currently the best animal model for this syndrome, displaying functional hyperdopaminergia and neurodegenerative phenotype leading to premature death in ~36% of the population. We used DAT-KO mouse as model for DTDS to explore the potential utility of a novel combinatorial adeno-associated viral (AAV) gene therapy by expressing DAT selectively in DA neurons and terminals, resulting in the rescue of aberrant striatal DA dynamics, reversal of characteristic phenotypic and behavioral abnormalities, and prevention of premature death. These data indicate the efficacy of a new combinatorial gene therapy aimed at rescuing DA function and related phenotype in a mouse model that best approximates DAT deficiency found in DTDS.

The key regulator of dopamine (DA) neurotransmission, the dopamine transporter (DAT) belongs to a family of plasma membrane transporters of solute carrier family 6 (*SLC6*), whose function is to uptake released DA from the extracellular space into the presynaptic neuron by coupling inward DA transport to movement of Na^+^ and Cl^−^1,2,3,4. DAT has an important homeostatic role in dopaminergic neurotransmission by controlling dynamics of both extracellular and intracellular dopamine^1^,^5^,^6^,^7^,^8^,^9^. Rare coding variants of the DAT gene have been identified in patients with ADHD, bipolar disorder and parkinsonism^3^,^10^. Kurian and coworkers identified a subgroup of parkisonism-dystonia patients with a loss-of-function mutation in the DAT gene SLC6A3^11^, whose spectrum was first assessed in pediatric patients^11^,^12^. This parkinsonian-like condition was recognized as an autosomal recessive disorder directly caused by impaired DAT functioning, and named Dopamine Transporter Deficiency Syndrome (DTDS); the clinical phenotypic continuum has been afterwards broadened to adult patients^13^, and recently new cases were reported^14^. Loss of DAT function was detected in most missense variants through *in vitro* functional studies^11^. The disease course affects motor and cognitive development and is associated with secondary medical complications and reduced life expectancy. Although several drug treatments were attempted, and new approaches are in development^15^, all of them were proven to be of little or no benefit to DTDS patients^11^,^12^.

Recent findings provided first insights in the neurobiology of DTDS, using a knockin *C. elegans* as a first *in-vivo* model for DTDS, expressing DTDS-mutated human DAT^16^. Nevertheless, the best available mammalian model for DAT deficiency is the DAT knockout (DAT-KO) mouse, which recapitulates the major clinical features of human DTDS patients. As seen in the majority of DTDS patients at early stages, DAT-KO mice display hyperkinesia starting from 3–4 weeks of age, and as in later stages of DTDS, more than 30% of these hyperactive mice progressively develop motor deficits and loss of hyperactivity, signs of striatal neurodegeneration and increased mortality^5^,^17^,^18^. The first signs of motor dysfunction are easily discernible when mice lose their typical hyperactivity, develop constant body clasps, display abnormal extension of the hindlimbs during a tail suspension test and a few days prior to death, also show pronounced weight loss, dorsal kyphosis and resting tremor^18^. It is known that persistent striatal hyperdopaminergia in DAT-KO mice results in an increase in the ratio of major extracellular DA metabolite homovanillic acid (HVA) over serotonin metabolite 5-hydroxyindolacetic acid (5-HIAA)^19^. Importantly, an increased HVA to 5-HIAA ratio was found in the cerebro-spinal fluid (CSF) of all tested DTDS patients and this measurement is currently considered as a distinctive diagnostic biomarker for DTDS^13^. Thus, despite different severities of the phenotype, all of the listed features render the DAT-KO mouse a suitable and readily available model for testing novel treatment approaches for pharmacoresistant DTDS, including gene therapy. We therefore used DAT-KO mice to test a novel combinatorial viral strategy aimed at restoring proper DAT expression and function by means of brain delivery of recombinant Adeno-Associated Viruses (rAAVs) engineered to deliver genes selectively to dopaminergic neurons. The combinatorial approach overcomes the small AAV packaging issue and simultaneously allows effective expression of the protein of interest in relatively large brain areas in specific neurons^20^,^21^.

Herein, we provide preclinical data demonstrating the efficacy of such novel target-specific mediated gene therapy that resulted in unprecedented rescue of the abnormalities in dopaminergic transmission, leading to the robust amelioration of major behavioral deficits and the prevention of the development of the neurodegenerative phenotype in our model. This work provides proof-of-principle evidence for a viable gene therapy aimed at restoring DA function selectively in dopaminergic neurons in an animal model of DTDS, thereby showing direct translational value to support the development of gene therapy approaches in human clinical trials for DTDS and potentially other genetic diseases related to DA dysfunction.

## Results

### Slc6a3 AAV delivery rescues DAT protein expression in nigrostriatal neurons of DAT-KO mice

**Figure 1:**
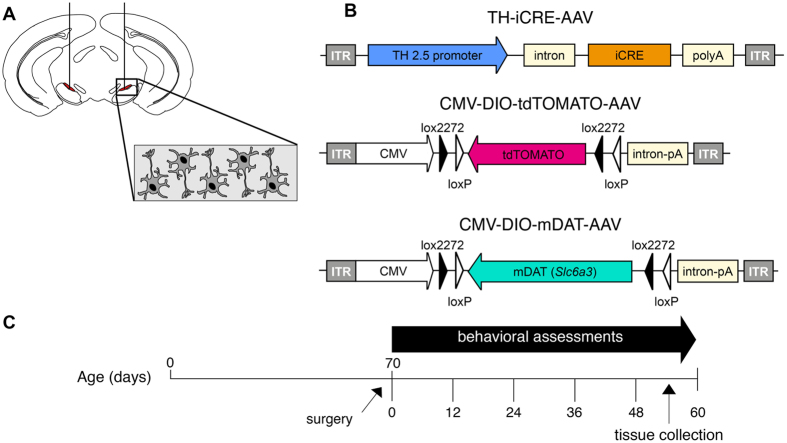
rAAV administration in Substantia Nigra. **(A)** Surgery. Adult DAT-KO mice underwent bilateral stereotaxic injection of AAV particles in the SN (coordinates: −3.2 mm AP, ±1.2 ML, −4.3 DV), where DA neurons originate and project to the striatum. **(B)** Dual combinatoral AAV strategy. TH-iCRE-AAV is used to confer the selective expression of improved CRE recombinase protein (iCRE – orange box) in DA neurons under control exerted by the 2.5 kilobase Rat Tyrosine Hydroxylase promoter (Rat TH – blue arrow). This construct was delivered in a 1:1 ratio with AAV constructs bearing an inverted sequence – indicated by a flipped (leftward) arrow – of the gene of interest under control of CMV promoter (CMV – white arrow). For the DAT-KO control group, the AAV construct bore the tdTOMATO gene sequence (CMV-DIO-tdTOMATO-AAV, magenta arrow), while for the DAT-KO treated group, the AAV bore the inverted sequence of the mouse Dopamine Transporter (mDAT) gene, depicted with a teal arrow (CMV-DIO-mDAT-AAV). **(C)** Experimental paradigm. Mice underwent surgery at postnatal day 70 (day 0) and were tested every 12 days.

**Figure 2:**
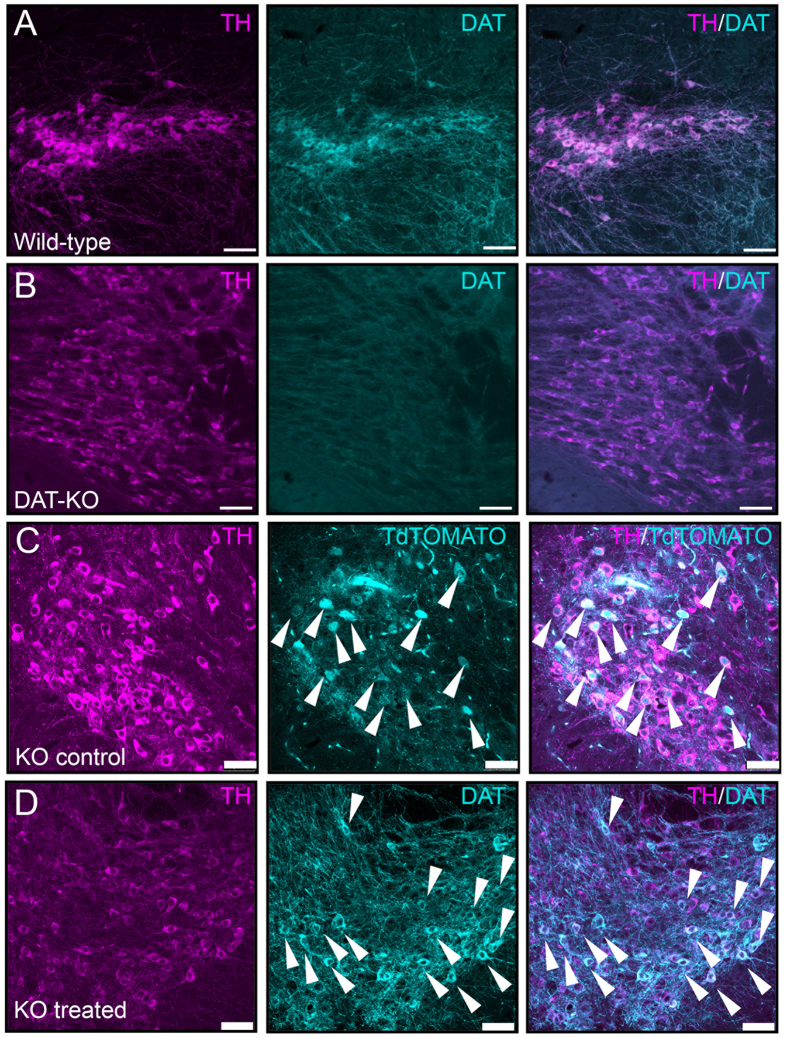
Slc6a3 gene delivery to SN mediates the rescue of DAT protein expression. Control confocal imaging of the Substantia Nigra for TH+ neurons as DA neuronal marker (magenta channel) and DAT positive cells (teal channel). **(A)** DAT signal is strongly evident in the case of WT animals while **(B)** absent in the DAT-KO mouse (scale bar 30 μm). **(C)** Pattern of cellular expression of the tdTOMATO protein with tyrosine hydroxylase (TH)+ cells in the SN (scale bar 50 μm). (**D)** Confocal imaging for the KO treated group displays co-localization of TH+ cells with DAT+ neurons (scale bar 50 μm).

Our primary goal was to develop a viable, stable and functional gene therapy approach aimed at reversing the molecular and behavioral abnormalities associated with the Slc6a3 deletion in DAT-KO mice. For this purpose, we used adult DAT-KO homozygote mice (age ~70 days), which were divided into DAT-KO control (KO control) and DAT-KO treatment (KO treated) groups. Animals were injected with viral constructs bilaterally in the SN (Fig. 1A) (−3.2 mm AP, ±1.2 ML, −4.3 DV) (adapted from Cao *et al*.^22^), where A9 dopaminergic cell bodies are located and send their axonal projections to the dorsal striatum (DS). As described in Materials and Methods, we took advantage of a recently published novel combinatorial dual AAV vector approach that allows specific dopaminergic expression while maintaining high expression levels of the gene of interest^21^. We attempted to achieve selective dopaminergic expression of tdTOMATO, a derivative of Red Fluorescent Protein (RFP), for the KO control group *or* mouse dopamine transporter (Slc6a3 – mDAT) for the KO treated cohort in the SN. In this combinatorial system, one rAAV incorporates either tdTOMATO or mDAT in a Double Inverted Open (DIO) reading frame driven by the CMV promoter (CMV-DIO-tdTOMATO-AAV and CMV-DIO-mDAT-AAV, respectively, Fig. 1B); in the absence of Cre recombinase protein, the genes are retained in a non-sense, inverted orientation. The second rAAV delivers a codon-optimized (“improved”) Cre-recombinase (iCre) protein under control of a rat Tyrosine Hydroxylase (TH) promoter (named as TH-iCre-AAV, Fig. 1B), which has been proven to drive protein expression selectively only in TH+ neurons in the SN, which are exclusively dopaminergic^21^,^23^. All constructs were packaged into pseudotyped AAV2/10 virus particles. TH-iCre-AAV was stereotaxically co-infused with CMV-DIO-mDAT-AAV in the DAT-KO treated group, or with CMV-DIO-tdTOMATO-AAV for the KO control group. Animals were tested for locomotor activity on pre-surgery day 0 and kept up to 48 to 60 days after surgery with monitoring tests performed every 12 days before sacrificing them to collect brain tissues (Fig. 1C). We performed immunostaining for the TH protein of coronal brain slices to visualize DA neurons in the SN. As a control, we performed immunostaining for TH and DAT in wild-type (WT) and DAT-KO naïve animals; the former displayed co-localization of TH+ and DAT expressing neurons (Fig. 2A), and the latter showed an absence of DAT expression (Fig. 2B) as expected from the genetic deletion of DAT previously described^5^. Confirmation of the injection site was achieved by analysis of KO control animals expressing tdTOMATO in this area. Robust expression of tdTOMATO that co-localized with TH+ DA neurons in the SN was observed (Fig. 2C). Most importantly, the Slc6a3 gene therapy approach in the treated KO group effectively rescued DAT expression selectively in TH+ dopaminergic neurons, as shown in co-localization studies in the SN (Fig. 2D).

### Slc6a3 gene therapy rescues striatal DAT expression, dopamine metabolism and uptake

**Figure 3:**
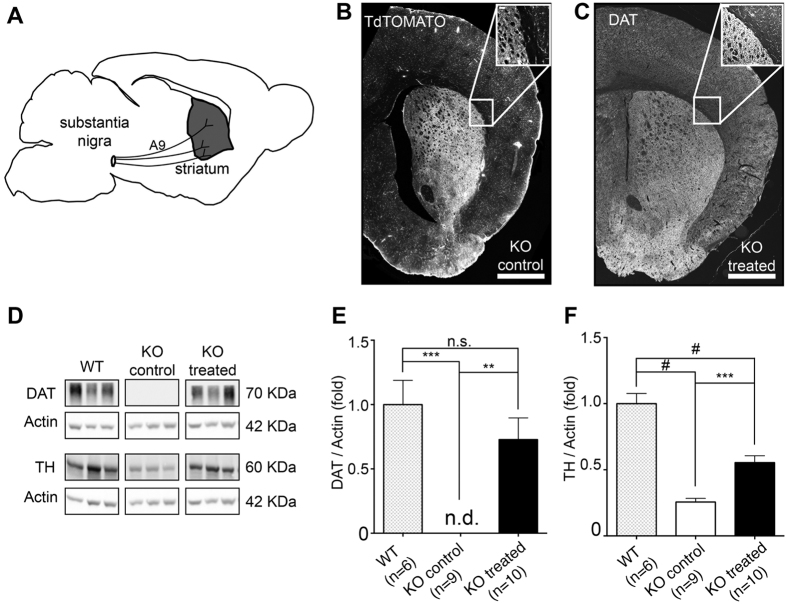
Analysis of major hallmarks of striatal dopaminergic signaling. (**A**) Schematic representation of the A9 nigrostriatal pathway originating from the SN and projecting to the dorso-lateral striatum. **(B)** Immunohistochemical detection of the tdTOMATO protein from the striatum of KO control animals injected with the CMV-DIO-tdTOMATO-AAV construct in the SN (scale bar 500 μm). **(C)** Widespread pattern of DAT expression in the striatum of KO treated animals, respectively (scale bar 500 μm). **(D)** Rescue of DAT protein expression in the striatum and restoration of TH expression levels in treated KO mice. The signal for mDAT was not detectable (n.d.) in the KO control group. **(E)** mDAT expression in the treated KO group was comparable to WT. **(F)** TH level is restored to ~50% of WT levels in treated KO animals compared to KO control mice. Actin bands displayed are from the same blot since the same samples were probed for either DAT or TH protein. For further details see Fig. S1. Data are expressed as the mean ± SEM. One-way ANOVA and Holm-Sidak *post-hoc* tests were used for multiple comparisons. ^#^p < 0.0001; ***p < 0.001; **p < 0.01; n.s. – not significant.

As briefly depicted, nigrostriatal A9 DA neurons are known to originate in the SN, sending their projection to DS (Fig. 3A). Confocal imaging of striatal slices showed robust expression of tdTOMATO protein in DS for the KO control group (Fig. 3B), and most notably widespread expression of DAT protein in the DS of KO treated animals (Fig. 3C), thus providing evidence of axonal transport of the protein of interest to the synaptic sites located in the striatum. Western blot analysis of striatal protein extracts further confirmed the restoration of DAT expression for the treated KO group (Fig. 3D,E) up to 75% of levels observed in WT littermates (one-way ANOVA F (2, 22) = 12.89, p = 0.0002; Holm-Sidak *post-hoc* p = 0.2073). No expression of DAT protein was detected in the striatal tissues obtained from the KO control group, as expected from previous studies on the DAT-KO mice^5^ (Fig. 3D,E). Slc6a3 gene therapy also resulted in the rescue of reduced striatal TH levels^19^ in the DAT-KO treated group (Fig. 3D–F) to approximately 50% of the WT levels, indicating a functional restoration in the level of rate limiting enzyme for DA synthesis (one-way ANOVA F (2,22) = 26.41, p < 0.0001; Holm-Sidak *post-hoc* p = 0.0002 *versus* KO control and p < 0.0001 *versus* WT).

Our next step was to assess the impact of Slc6a3 gene therapy on dopaminergic homeostasis in the striatum, which was proven to be severely affected by the lack of DAT^5^,^18^,^19^,^24^,^25^,^26^. Disruption of DAT-mediated inward transport due to the lack of DAT in mutant mice results in remarkable changes in the status of the striatal dopaminergic system, most notably manifesting as disrupted clearance of released dopamine, the extremely prolonged extracellular lifespan of DA, the depletion of intraneuronal DA stores, alterations in DA metabolism and decreased levels of TH^19^,^25^. Slc6a3 gene therapy reversed all these manifestations.

**Figure 4:**
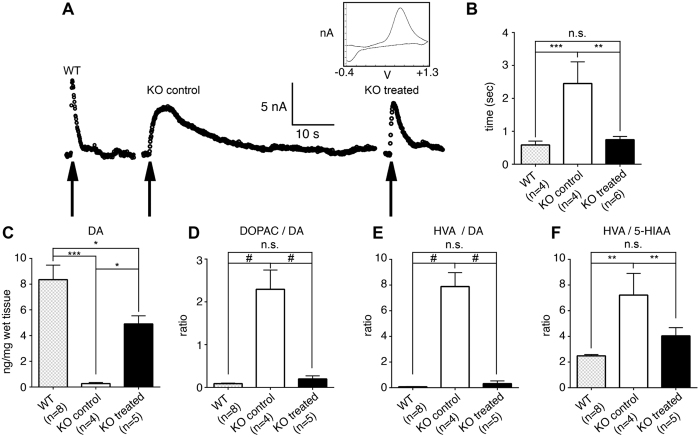
Slc6a3 gene therapy rescues dopamine uptake, storage and metabolism in the striatum. **(A)** FSCV measurements of DA release in striatal slices. Representative traces of individual measurements from animals in each experimental group and representative DA voltammogram (current/voltage) are displayed. (Inset). Dopamine release was evoked by a single electrical pulse (400 μA, bipolar pulse), applied at the time indicated by the arrow. Extracellular DA was sampled every 100 ms and the rising portion of the curve represents release, whereas the falling portion represents clearance of DA. **(B**) Difference in DA clearance rate in striatal slices among WT, KO and KO treated mice as measured by DA half-life. **(C)** Effects of Slc6a3 gene delivery on striatal tissue levels of DA: treated KO animals have partially restored DA levels **(D)** DOPAC/DA ratio was rescued in treated KO animals as well as **(E)** HVA/DA ratio. **(F)** Increased HVA/5-HIAA ratio in the untreated KO group was rescued in treated KO mice. Data are expressed as the mean ± SEM. One-way ANOVA and Holm-Sidak *post-hoc* tests were used for multiple comparisons. ^#^p < 0.0001; ***p < 0.001; **p < 0.01; *p < 0.05; n.s. – not significant.

Fast Scan Cyclic Voltammetry (FSCV) was used to measure electrically-evoked efflux of DA in the striatal slices from WT, untreated KO and treated KO mice. DA release was evoked by a single electrical pulse (400 μA, biphasic pulse) and extracellular DA was sampled every 100 ms. As demonstrated previously^5^,^19^, untreated KO mice have a remarkably reduced clearance rate of DA in comparison to WT controls as reflected by increased DA half-life measures (Fig. 4A,B). In treated KO animals, we achieved significant rescue of the DA clearance rate, which was comparable to the WT level following Slc6a3 gene therapy (Fig. 4A,B). In fact, DA half-life was significantly decreased in KO treated animals (one-way ANOVA F(2,16) = 12.82, p = 0.0005, Holm-Sidak *post hoc*, p = 0.0015 *versus* KO control, and p = 0.6426 *versus* WT animals), thus providing proof that DA reuptake into the pre-synaptic terminals was reestablished after Slc6a3 gene therapy (Fig. 4A,B).

HPLC analysis of tissue monoamines and metabolites levels showed that Slc6a3 gene therapy restored striatal tissue DA content – which is known to be depleted 20-fold in DAT-KO mice^19^ – to ~70% of WT levels (one-way ANOVA F(2,14) = 15.42, p = 0.0003, Holm-Sidak *post-hoc*, p = 0.0234 *versus* KO control and p = 0.0242 *versus* WT), thus providing evidence of restoration of proper DA storage (Fig. 4C). In addition, this treatment fully rescued the ratios of striatal DA metabolites DOPAC (3,4-dihydroxyphenylacetic acid) to DA (one-way ANOVA F(2,14) = 160,6, p < 0.0001; Holm-Sidak *post-hoc*, p < 0.0001 *versus* KO control and p = 0.375 *versus* WT littermates. Figure 4D) and HVA (Homovanillic Acid) to DA (one-way ANOVA F(2,14) = 345,5, p < 0.0001; Holm-Sidak *post-hoc*, p < 0.0001 *versus* KO control and p = 0.424 *versus* WT littermates. Figure 4E) used as indexes of DA turnover as previously described^27^. Absolute levels of striatal monoamines and metabolites are presented in Table S1.

One additional neurochemical feature common for DAT-KO mice and DTDS patients is the elevated ratio of the extracellular DA metabolite HVA to a major serotonin metabolite, 5-hydroxyindolacetic acid (5-HIAA)^19^, as it is demonstrated in KO control mice in this study. Full rescue of the HVA/5-HIAA ratio was observed for treated DAT-KO mice to levels comparable to those observed in WT mice (one-way ANOVA F (2,14) = 9.77, p = 0.0022; Holm-Sidak *post-hoc* p = 0.414 Fig. 4F). Such rescue of the HVA/5-HIAA ratio indirectly indicates the normalization of extracellular DA dynamics after the rescue of striatal DAT expression driven by nigral delivery of the combination of TH-iCre-AAV with CMV-DIO-mDAT-AAV constructs. The ratio of the DA metabolite HVA to 5-HIAA was found to be augmented in cerebrospinal fluid (CSF) of DTDS patients compared to age-matched healthy patients, indicating increased striatal DA turnover at the extrasynaptic level due to the hyperdopaminergic state induced by the absence of DAT activity^11^,^13^. Thus, these data provide the first proof-of-principle evidence that Slc6a3 gene therapy in adult mice is able to rescue this fundamental feature and essential biomarker of DTDS.

### Rescue of hyperactivity and other behavioral phenotypes in DAT-KO mice

**Figure 5:**
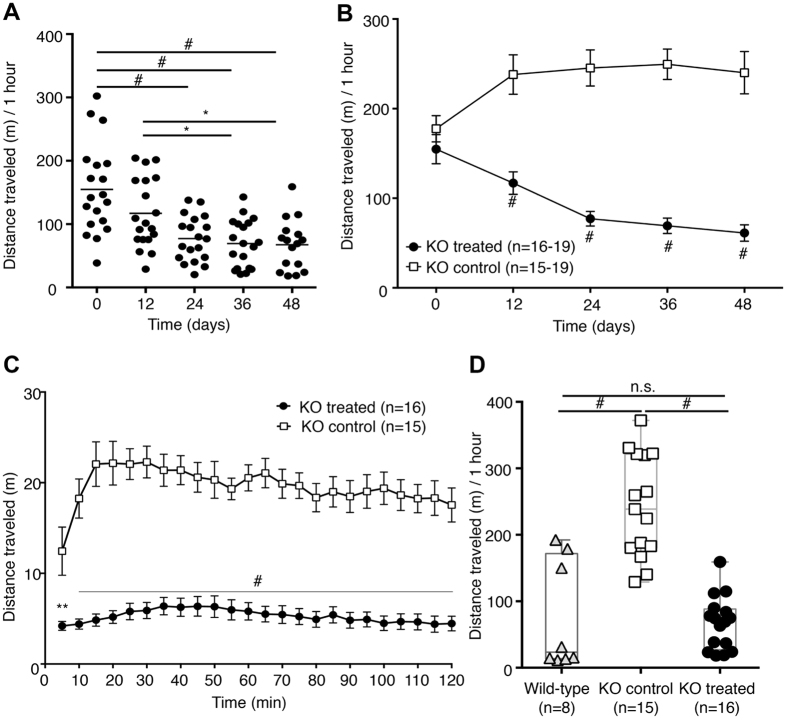
Slc6a3 gene therapy reduces hyperlocomotion in DAT-KO mice. **(A)** DAT-KO treated mice showed reduction in total distance traveled in 1 hour in the open field starting at Day 24 after surgery. **(B)** When compared to KO control animals, treated KO mice displayed lower total distance starting at Day 12 after surgery until Day 48 (Bonferroni *post-hoc*, p < 0.0001). **(C)** Dynamics of locomotor activity in 5-minute intervals depicting activity on Day 48. **(D)** rAAV-based rescue of total distance traveled for treated KO animals on Day 48, to levels that did not differ from those of WT littermates naïve to the test. Data are expressed as the mean ± SEM. One-way ANOVA + Holm-Sidak *post-hoc* and two-way ANOVA + Bonferroni *post-hoc* tests were used for multiple comparisons. ^#^p < 0.0001; ***p < 0.001; **p < 0.01; *p < 0.05; n.s – not significant.

DAT-KO mice display prominent hyperactivity when exposed to a novel environment^5^. Slc6a3 gene therapy treatment significantly decreased the total distance travelled in locomotor boxes (one-way ANOVA, F(4,87) = 10.76 p < 0.0001, Holm-Sidak *post-hoc* p < 0.0001 on Day 24, 36 and 48 Fig. 5A). When compared to the KO control group, the distance travelled by treated DAT-KO mice was significantly different from Day 12 up to Day 48 after surgery (two-way ANOVA F(4,172) = 9.004, p < 0.0001; Bonferroni *post-hoc*, p < 0.0001 Fig. 5B). The analysis of the dynamics of locomotor activity presented as total distance travelled in the open field test performed on Day 48 revealed that Slc6a3 gene therapy decreased activity after 5 minutes from exposure to the novel environment for treated KO mice compared to the KO control group (two-way ANOVA F(23,667) = 2.827, p < 0.0001 Bonferroni *post-*hoc, p < 0.0001 Fig. 5C). When the cumulative distance traveled in the open field in 1 hour by either the KO control or the treated KO group was compared to those of test-naïve WT littermates, no difference was observed between treated KO and WT mice (one-way ANOVA, F(2,36) = 31.96, p < 0.0001; Holm-Sidak *post-hoc*, p = 0.7680 Fig. 5D). These results suggest that gene therapy-based rescue of striatal DAT expression in DA neurons in DAT-KO mice fully rescued activity to WT levels. Furthermore, the Slc6a3 gene therapy approach further normalized stereotyped activity, as demonstrated in Fig. S1A and S1B.

**Figure 6:**
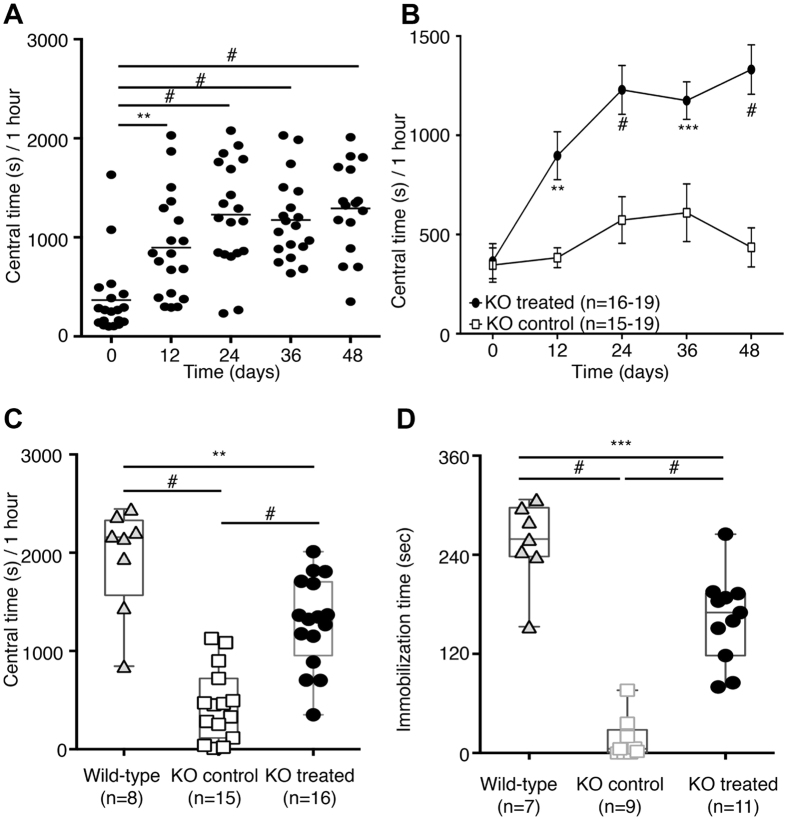
Other behavioral effects of Slc6a3 gene therapy in DAT-KO mice. **(A**) Slc6a3 gene therapy induces an increase in the time spent in the central compartment of the locomotor boxes starting from 12 days from surgery and remained stable from Day 24, 36 and 48 in treated KO animals. **(B)** A dramatic progressive increase of normal exploratory behavior in treated KO animals compared to KO controls, expressed as time spent in the central area of the open field. **(C)** Slc6a3 gene therapy rescued the central time in DAT-KO mice to ~65% of WT performance 48 days after treatment. **(D)** Performance of treated and control DAT-KO mice in the Porsolt forced swim test. Slc6a3 gene therapy restored normal swimming behavior in treated KO animals compared to the KO control group. Data are expressed as the mean ± SEM. One-way ANOVA+ Holm-Sidak *post-hoc* and two-way ANOVA+ Bonferroni *post-hoc* tests were used for multiple comparisons.^#^p < 0.0001; ***p < 0.001; **p < 0.01; n.s – not significant.

As previously described^17^, DAT-KO mice displayed a perseverative pattern of locomotor activity known as ‘thigmotaxis’ when tested in an open field^28^. Interestingly, Slc6a3 gene therapy also affected this behavior, which manifested in DAT-KO mouse as a decreased time in the center due to extreme activity along the perimeter of the cage. In treated KO animals on Day 12 after surgery (one-way ANOVA F(4,87) = 12.28, p < 0.0001, Holm-Sidak *post-hoc*, p = 0.0052), the central time was doubled compared to Day 0/pre-surgery and a mean three-fold increase for this parameter was observed from Day 24 up to Day 48 (Holm-Sidak *post-hoc* p < 0.0001) (Fig. 6A). When compared to the KO control group, the time spent in the central compartment showed a 2-fold increase in treated KO animals on Day 12 from surgery, and reached a 3-fold increase 48 days after surgery (two-way ANOVA F(4,171) = 3.869, p = 0.0049; Bonferroni *post-hoc*, p = 0.0013 Fig. 6B). In particular, the cumulative central time measured in 1-hour sessions in treated KO animals was rescued to ~65% of WT mouse levels (one-way ANOVA F(2,36) = 31.96, p < 0.0001; Holm-Sidak *post-hoc*, p = 0.0016 Fig. 6C). Locomotor activity patterns in treated KO, KO control and WT groups are presented in Fig. S2A.

DAT-KO mice are also known to dramatically differ from WT controls in the Porsolt forced swim test^29^. During the forced swim test, KO control mice engaged in more pronounced swimming without developing an adaptive strategy^30^ in comparison to WT mice. Slc6a3 gene therapy reduced swimming time in treated KO mice, which reached ~65% activity of WT animals performance levels (one-way ANOVA F(2,24) = 56.63, p < 0.0001; Holm-Sidak *post-hoc*, p < 0.0001 Fig. 6D). Taken together, these data provide strong support of reversal – and in several cases complete rescue – of DA-dependent behaviors after Slc6a3 gene therapy treatment in DAT-deficient mice.

### Slc6a3 gene therapy prevents the neurodegenerative phenotype in DAT-KO mice

**Figure 7:**
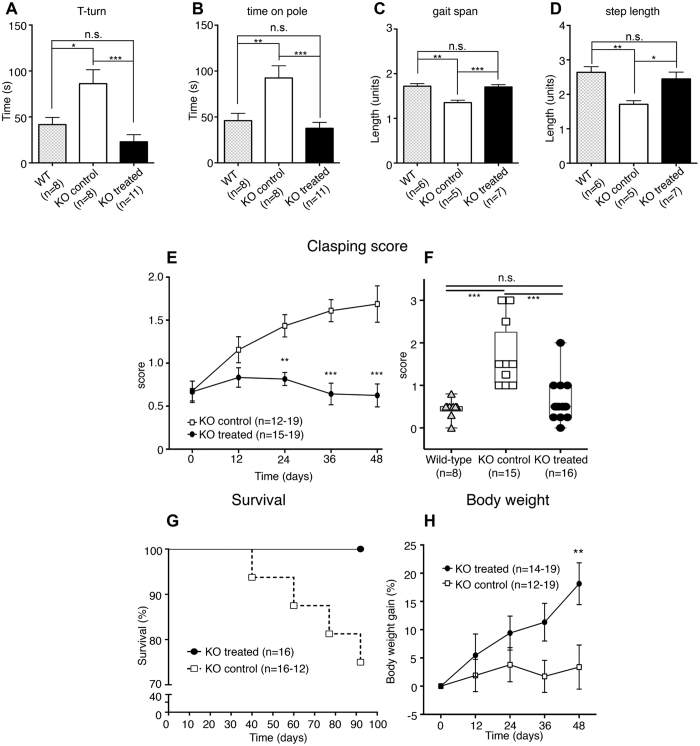
Slc6a3 gene therapy prevents motor deficits and the neurodegenerative phenotype in DAT-KO mice. Rescue of motor performance in the pole test measured as **(A)** time to turn from an upside down position in treated KO animals (T-turn). **(B**) Decrease in the time treated KO descend from the pole when differs from the performance of WT mice. Rescue of **(C)** gait width and stride length **(D)** in treated KO animals compared to KO controls after Slc6a3 AAV delivery. **(E)** The prevention of clasping behavior in the tail suspension test for the treated KO cohort. **(F)** Total rescue of clasping behavior at the tail suspension test, in which treated KO animals demonstrated scores similar to those of WT mice. **(G)** Mantel-Cox survival curve (p = 0.0353) depicting the absence of mortality in KO control animals (n = 16) compared to treated KO mice (n = 16–12). **(H)** Monitoring of body weight increase. KO treated animals display higher body weight gain 48 days after AAV delivery (Two-way ANOVA F(1,171) = 13.56, Bonferroni *post-hoc* test, p = 0.003). Data are expressed as the mean ± SEM. One-way ANOVA+ Holm-Sidak *post-hoc* and two-way ANOVA+ Bonferroni *post-hoc* tests were used for multiple comparisons. ***p < 0.001; **p < 0.01; *p < 0.05; n.s – not significant.

We further evaluated whether Slc6a3 gene therapy could have possible effects on the development of the neurodegenerative phenotype and may affect survival rate. The DAT-KO mouse neurodegenerative phenotype has been thoroughly investigated at both the behavioral and molecular levels. It is known that even the general population of DAT-KO mice displays minor motor impairments in several behavioral tests. Particularly, DAT-KO mice significantly differ from WT mice in the pole test, which is commonly used to assess motor coordination in animal models of parkinsonism^31^,^32^,^33^ and measures the ability to properly coordinate limb movement, particularly the hindlimbs^17^. The pole test measures the ability of a mouse to turn upside-down when posed on the top of a wooden pole (‘T-turn’) and the time spent to coordinately walk down the pole (‘Time on pole’). When compared to WT mice, KO control animals displayed a 2-fold increase for both parameters, thus confirming previous observations^17^. Slc6a3 gene therapy completely rescued both T-Turn (one-way ANOVA F(2,24) = 9.937, p = 0.0007; Holm-Sidak *post-hoc* test p = 0.0005 *versus* KO control; p = 0.2033 *versus* WT. Figure 7A) and Time on pole (one-way ANOVA F(2,24) = 10.11, p = 0.0007; Holm-Sidak *post-hoc* p = 0.0007 *versus* KO control; p = 0.5190 *versus* WT. Figure 7B) performances for the treated KO group to levels comparable to their WT littermates.

Furthermore, we analyzed footprint patterns as previously described^18^ at ~50 days post-rAAV delivery. Deficits in both gait width and stride length were observed in the KO control group compared to WT mice, thus confirming the development of gait abnormalities associated with striatal hyperdopaminergia^18^. In the case of treated KO mice, Slc6a3 gene therapy rescued both motor parameters of the footprint analysis for gait span (one-way ANOVA (F (2,15) = 13.16, p = 0.0005; Holm-Sidak *post-hoc* test p = 0.0008; p = 0.7314 *versus* WT. Figure 7C) and step length (one-way ANOVA (F (2,14) = 7.319, p = 0.0061; Holm-Sidak *post-hoc* p = 0.0177; p = 0.4301 *versus* WT. Figure 7D) to levels observed for WT mice. The typical footprint patterns of control KO, treated KO and WT mice are shown in Fig. S2B.

The most prominent motor deficits of DAT-KO mice are manifested in the tail suspension test as clasping behavior, which is known to be associated with striatal dysfunction^18^. All animals were monitored daily after surgery, and behavior during the tail suspension test was recorded from day 0 and every 12 days post-surgery, as previously described^18^,^34^. DAT-KO treated mice did not show increased progression of clasping scores during the 15-second tail suspension test, while KO control animals exhibited higher clasping and dorsal kyphosis scores starting from day 24 onwards when compared to the treated KO group (two-way ANOVA, F(4,160) = 5.631, p = 0.0003; Holm-Sidak *post-hoc* p = 0.0016 Day 24; p < 0.0001 for Day 36 and Day 48 Fig. 7E). KO control animals developed dorsal clasping as depicted (Fig. S2C). Slc6a3 gene therapy fully rescued clasping behavior in treated KO mice at 48 days post-surgery to the levels observed in naïve WT littermates (one-way ANOVA F(2,37) = 21.87, p < 0.0001; Holm-Sidak *post-hoc*, p = 0.3549 *versus* WT mice); their score was significantly different compared to the KO control group (Fig. 7F).

Finally, as discussed above, a subpopulation of DAT-KO mice, estimated to be ~36%, is known to develop more pronounced motor deficits and a severe neurological phenotype, eventually leading to death^18^. It is believed that this phenotype might be caused by excessive stimulation of dopamine receptors in striatal neurons, leading to the degeneration of MSNs^18^. Thus, we investigated whether Slc6a3 gene therapy could affect the survival rate of DAT-KO mice. In fact, Slc6a3 gene therapy prevented mortality in the treated KO animals compared to the KO control cohort (Mantel-Cox test, p = 0.0353. Figure 7G), thus providing direct evidence of the effectiveness of the rAAV approach in mouse model of DTDS. It is notable also that the general population of DAT-KO mice demonstrate lower weight compared to WT or heterozygous mice^5^. As another indication of normalized dopamine transmission following gene therapy, treated KO animals gained more weight compared to the KO control group (p = 0.003), as measured 48 days after surgery (Fig. 7H). Taken together, these data provide strong proof-of-principle evidence of the prevention of the development of the neurodegenerative phenotype in DAT-KO animals by a combinatorial rAAV-based gene therapy strategy.

## Discussion

DTDS is an autosomal recessive disorder caused by severe loss-of-function mutations in the SLC6A3 gene^35^. In most cases, mutations found in patients affected by DTDS led to loss-of-function of the DAT protein, often resulting in the complete lack of residual DAT activity^11^,^13^. Several drug administration protocols have failed in providing long-term beneficial effects^11^ and our group was the first to provide *in-vivo* insights of syndrome neurobiology using *Caenorhabditis elegans* to generate knockin models expressing human mutated DAT from patients displaying either “typical” DTDS or “atypical” form of the syndrome^15^.

DAT-KO mice can be used as a model of DTDS because they share a genetic background and the absence of DAT functionality, accompanied by a set of phenotypic features that are strikingly reminiscent of DTDS symptomatology, including hyperkinesia at early stages, followed by the progression of motor impairment, hypokinesia/dyskinesia and dorsal kyphosis^5^,^17^,^18^. This severe neurological phenotype is also associated with the emergence of markers of striatal neurodegeneration and increased mortality (~36% of the homozygote population)^18^. The phenotype shown by this portion of the homozygote population progresses in several days to weeks and is accompanied by evident weight-loss, leading to death^18^. As previously published, DTDS patients may develop a plethora of neurological symptoms similar to those displayed by DAT-KO mice.

Here, we demonstrate the efficacy of a novel dual combinatorial AAV-based gene therapy approach aimed at the restoration of native Slc6a3 DAT gene expression, a strategy that can provide basis for development of clinical trials for DTDS. In particular, we observed the effective rescue of the dopaminergic dysfunction of DAT-KO mice by means of a combinatorial AAV approach that uses the first AAV vector (TH-iCre) to allow selective expression of the Cre-protein in dopaminergic neurons under the control of the rat TH promoter^23^. As recently published^21^, such Cre expression is able to trigger cell-specific gene expression of the second AAV construct, which is under control of the strong CMV promoter, allowing robust expression of the protein of interest (DAT protein *or* tdTOMATO as control in this study). The second construct is present in a Double-Inverted Open (DIO) reading frame conformation, and therefore, the protein of interest is only expressed in cells in which the Cre protein is present. Our aim is to selectively express the DAT protein in dopaminergic neurons of the SN to anatomically restrict AAV’s action on A9 DA neurons projecting to DS, therefore acting on the primary DA area involved in motor control, whose dysfunction has been thoroughly characterized in our mouse model^17^,^18^,^24^,^26^. In this regards, ectopic DAT expression in non-DA striatal neurons (which will likely occur using non-selective AAV-mediated expression of DAT) has been proven to cause neurotoxicity in non-DA cells that do not have the complete cellular machinery to properly store and recycle DA, leading to motor dysfunction, body weight loss and lethality^36^. Finally, AAV-tdTOMATO gene reporter was used instead of Hemagglutin-tagged DAT gene because it has been recently demonstrated that this tagging impairs DAT expression *in vivo,* partially due to improper trafficking of the transporter^37^. It is notable that previous attempts to express WT DAT using the AAV virus resulted in non-selective expression of N-terminal hemagglutinin tagged form of the transporter in mutant mice that have functional but cocaine-insensitive DAT, with minimal behavioral phenotypes^38^.

Our Slc6a3 gene therapy drives robust expression in midbrain dopaminergic neurons, which was also detected at the synaptic sites located in the striatum. Most importantly, this treatment significantly reversed dramatic alterations in major parameters of striatal dopaminergic transmission described earlier in DAT-KO mice and recapitulated in the present study in control KO animals^19^. Most strikingly, the restoration of DAT expression increased clearance rate, decreased extracellular DA half-life and reversed the profound depletion of intracellular DA stores as reflected by the total tissue levels of DA, along with the rescue of dopamine turnover rate as shown by a higher total tissue metabolite (either DOPAC or HVA) to DA ratio. Another indication of normalized presynaptic dopaminergic homeostasis is reflected by the normalization of TH levels, known to be suppressed in DAT-KO mice due to the overactivity of the enzyme caused by the absence of newly transported DA in the cytoplasm that normally inhibits TH activity^7^,^19^. It has been hypothesized that DAT loss-of-function mutations in DTDS could lead to the activation of negative feedback on TH protein levels by means of DA overstimulation of D2/D3 autoreceptors^39^,^40^; thus, such a reversal may have functional ameliorating implications. Taken together, these data demonstrate the effective rescue of striatal DAT expression and function at either the neuronal or molecular level, as evidenced by the normalization of major neurochemical and signaling parameters known to be dramatically affected in DAT-KO mice. It is important to note that an increased HVA/5-HIAA ratio in cerebrospinal fluid, reflecting altered brain DA signaling, has also been found in all DTDS patients whose samples were available for analysis^11^,^12^,^13^. Strikingly, Slc6a3 gene therapy was able to fully rescue the similarly altered HVA/5-HIAA ratio in DAT-KO mice, thus directly validating this approach for future translational studies in humans.

The most evident effect of Slc6a3 gene therapy was reflected in the remarkably altered overt behavioral phenotype of treated mice that showed a reversal of both the hyperactivity and perseverative pattern of locomotion characteristic of this model of hyperdopaminergia. Furthermore, the development of motor abnormalities leading to death of affected animals was completely prevented. These motor abnormalities are presumably caused by ongoing neurodegenerative processes in the dopamine-receptive GABAergic medium-spiny neurons due to overactivation of dopamine receptors and related signaling events^18^.

Taken together, these data provide strong proof-of-concept evidence that a single local injection of viral constructs is highly efficacious and sufficient to provide the functional recovery of DAT expression and function in adult DAT deficient mice, thus defining a new window of opportunity for the treatment of DTDS.

However, because the majority of DTDS patients have early onset in childhood, it is important to demonstrate the efficacy of our approach in neonate animals developing brain, as dopaminergic pathways development starts during early fetal stages^41^ and continues throughout peri- and post-natal phases^42^,^43^. Nevertheless, some patients either display adult onset or reach the adult stage after childhood manifestation^13^, and therefore our approach already provide possibility for clinical development.

Obvious concerns related to scalability of our findings in the developing human brain that also has longer ontogenesis and lifespan compared to mouse brain need to be addressed before this approach may be applied in clinical practice for long-term efficacy. On a positive note, gene therapy approaches are being currently explored and showed some effectiveness in clinical trials as an alternative to pharmacological treatment of neurodegenerative diseases, such as PD^44^,^45^,^46^,^47^; in one open label-study, gene therapy was proven to be tolerable and safe in patients with Parkinson’s Disease followed for 2 years^46^, and applications in pediatric patients lead to positive outcomes, such is the case of Aromatic Amino Acid Decarboxylase (AADC) deficiency^48^,^49^. In most of these trials a recombinant AAV2 serotype was used for human applications and more recently characterized AAV serotypes, particularly AAV10, are proving effective at transducing larger brain regions in non-murine/larger species^20^, as in our proof-of-principle study the constructs were pseudotyped and packaged into AAV2/10 serotype particles.

In summary, we established the use of DAT-KO mice as a model to understand pathological processes and develop novel effective treatments for the recently discovered DTDS. Furthermore, we explored the effectiveness of a newly developed targeted combinatorial Slc6a3 gene therapy strategy that resulted in the selective and robust expression of functional DAT in dopaminergic neurons, leading to efficacious recovery of major neurochemical, molecular and behavioral phenotypes related to DAT deficiency. Remarkable rescue of motor dysfunction, neurodegenerative phenotype and decreased mortality was observed, and the treatment proved to be safe and tolerable up to ~100 days after AAV delivery. Development of the motor dysfunction and neurodegenerative phenotypes that sporadically affect part of the DAT-KO homozygous population^17^,^18^,^29^ was fully prevented.

This proof-of-principle study therefore validates the feasibility of the novel AAV-based technology for development of therapeutic approach for DTDS patients. Furthermore, these data indicate that a similar AAV-based combinatorial approach might have wide applications in gene therapy studies, paving the way to the development of effective cell-selective AAV-based strategies for pre-clinical and clinical application in other disorders.

## Methods

### Subjects

The generation of C57BL/129SvJ DAT knockout mice was previously described^5^, and these animals were intercrossed for >10 generations. Mice were housed 2–3/cage until surgery and maintained under standard lab conditions (12 h light/dark cycle, 21 ± 1 °C and 40–70% humidity) with food and water provided *ad libitum*. After surgery, mice were single-housed in environmentally enriched cages, in order to reduce the possibility of injuries due to stitch removal. A total 54 DAT-KO homozygous mice of both sexes (26 females/28 males), and 20 WT mice (8 females/12 males) were used in these experiments. Animals underwent stereotaxic surgery at ~70 post-natal days and were tested afterwards every 12 days until 48 days. All experiments were conducted during the light phase. Only animals that fully recovered from surgery were included in this study, which always started with the measurement of locomotor activity 12 days after surgery, in order for mice to fully recover. Animals that were not able to recover from the AAV injection surgery (3 in total) were not included in any of the reported results.

### Genetic constructs

The TH-iCRE-pAAV construct has been described previously^21^. Briefly, this plasmid consists of a 2.5-kb segment of the rat TH promoter, which predominantly drives expression in TH neurons, including those found in the midbrain and locus coeruleus^21^,^23^, and Cre recombinase. The FLEX-mDAT-pACP plasmid consists of a FLEX cassette, into which the mouse dopamine transporter (mDAT) cDNA was inserted in the reverse orientation. pACP is a plasmid consisting of two AAV2 inverted terminal repeats flanking a CMV promoter, multiple cloning site, and a combined intron/polyA sequence derived from SV40. A FLEX switch^50^, which is equivalent to the double floxed inverted open reading frame (DIO), was synthesized (Genscript) and inserted into the multiple cloning site of pACP to generate FLEX-pACP. The mouse DAT gene (Slc6a3) was obtained from the Mammalian Gene Collection (cDNA clone MGC:58286 IMAGE:6588897) in pDNR-LIB (Fisher Scientific Cat. No. MMM1013-202842662). The mDAT cDNA was amplified using the following primers: 5′-ACGCGTCGACGCCACCATGAGTAAAAGCAAATGCTCC-3′ and 5′-GATCTCTAGATTATTACACCAACAGCCAATGGCGCAGCG-3′. The product was digested with SalI and XbaI and inserted in the inverse orientation into the XbaI and XhoI sites of the FLEX-pACP plasmid.

### AAV packaging

Both TH-iCre-AAV2/10 and FLEX-mDAT-AAV2/10 were packaged using the standard triple transfection protocol in HEK-293 cells to create recombinant pseudotyped AAV2/10 virus^51^. Briefly, either of the AAV plasmids described above were co-transfected with a plasmid that provides the AAV2 replicase and AAV10 capsid genes and a third plasmid, pHelper, that contains adenoviral helper functions. AAV2/10 virus is highly neurotrophic, is easily titrated, and has previously been used by our laboratories to drive high levels of expression in both extremely small and large brain regions of both rats and mice, primarily by adjusting volume^52^,^53^,^54^,^55^. Viruses were titered using quantitative PCR to approximately 10^13^ vector genome copies per ml.

### Stereotaxic surgery

All surgical procedures were performed under general anesthesia using an Isoflurane/O_2_ mixture flow at a ratio of 2.5:2.5. Mice were placed in a stereotaxic frame (Stoelting) and vector solutions were injected using a glass capillary (outer diameter of 250 um). 0.5 μl of TH-iCRE-AAV either mixed with 0.5 μl CMV-DIO-mDAT-AAV or 0.5 μl CMV-DIO-tdTOMATO-AAV was infused at a rate of 0.2 μl/min, and the glass capillary was left in place for an additional 10-min period before it was slowly retracted. Injections were carried out bilaterally targeting the SN at the following coordinates (flat skull position): antero-posterior (AP) – 3.2 mm, medio-lateral (ML) ±1.2 mm, dorso-ventral (DV) 4.3 mm, below the dural surface as calculated relative to bregma, according to the stereotaxic atlas by Paxinos and Watson^56^. 27 animals received TH-iCRE-AAV + CMV-DIO-mDAT-AAV (KO treated – 15 males/12 females) and 27 animals were injected with TH-iCRE-AAV + CMV-DIO-tdTOMATO-AAV (KO control – 13 males/14 females). After surgery, all animals were housed singly in a cage with enriched environment and checked daily for 1 week for general health status without any manipulation, and left undisturbed until post-surgery day 12, when the first locomotor activity test was performed.

### Locomotor activity

Locomotion was evaluated as described^57^ using an automated Omnitech Digiscan apparatus (AccuScan Instruments, USA) under illuminated conditions. The apparatus included four open field monitors, which each consisted of a set of 16 light beams arrayed in the horizontal X and Y axes. The hardware detected beam breaks by the animal, which allowed the software to determine the location of the mouse in the cage. Cages were divided into four compartments (20 cm × 20 cm). Animals were tested individually for defined periods in 5-min intervals. The total distance was expressed in terms of meters traveled by the animal, while the central time expressed the time spent in the central compartment. Stereotypy was represented by the count of the number of repeated beam breaks patterns that occurred during the observation period. Locomotor activity was recorded for 60 minutes before stereotaxic surgery and every 12 days from surgical intervention until 48–60 days.

### Pole test

The pole test was performed as described elsewhere^17^, with minor modifications. The mouse was placed head upward on top of a wooden rough-surfaced pole (diameter 1 cm, height 70 cm). Each mouse was habituated to the pole for two days prior to testing, then allowed to descend five times in a single session. Two parameters were measured. First T-total: total time spent by the mouse to reach the floor with the four paws; secondly, T-turn: time spent by the animal to completely turn downwards during descent from the pole. If the mouse was unable to completely turn downwards or fell or slipped down from the pole, the max default value of 120 seconds was assigned.

### Tail suspension test and footprint analysis

Tests were performed as described^18^. In the tail suspension test, mice were habituated to the test room 1 hour prior to testing. Mice were held by the tail for 15 seconds and clasping behavior was assessed. The clasping score was equal to 0 if no clasping was observed during the test time, 1 if abnormal extension of the hindlimbs was noticed, 2 if the mouse showed partial clasping, and 3 when the mouse displayed firmly established clasping. For footprint analysis, each paw of the mouse was dipped in a different color of edible paint and the mice were habituated for one day to the testing chamber, a cardboard tunnel with a wide filter paper pad on the floor. On the test day, the footprint strips were collected, scanned and analyzed with ImageJ software. Analysis was performed only if mice were able to walk at least 3 steps without stopping. Tests were scored by an experimenter blind to treatment conditions.

### Porsolt test

The Porsolt test was carried out as previously described^29^. Animals were forced to swim individually in a vertical 18-cm glass cylinder, diameter 15 cm, and containing 13.5 cm of water maintained at ~25 °C. After 6 min in the water, they were removed and allowed to dry under a heating lamp for 10 minutes. The total duration of immobility was measured. An animal was judged to be immobile whenever it remained floating in the water in a slightly hunched but upright posture, the head just above the water.

### Immunohistochemistry

Mice were pre-anesthetized with isoflurane and anesthetized with urethane at a dose of 2 g/kg. Animals were transcardially perfused with 20 ml/min of ice-cold saline (0.9% w/v) for 5 minutes, followed by double cycles of 20 ml/min and 10 ml/min of ice-cold paraformaldehyde (4% w/v in 0.1 M phosphate buffered saline - PBS) for 5 minutes each. Brains were removed, post-fixed overnight in 4% paraformaldehyde and cryoprotected overnight in 0.5 M sucrose in 0.1 M PBS before sectioning on a freezing microtome (Leica). Coronal sections were collected from −3.88 mm to −2.30 mm antero-posteriorly in 12 series at a thickness of 30 μm. Free-floating slices were quenched in sodium borohydride (0.5% in PBS) for 10 minutes and rinsed 3 times with PBS. Blocking (10% NGS, 0.75% bovine serum albumin [BSA], 0.1% Triton-X) was carried out for two hours. Immunohistochemical staining was performed on free-floating sections using antibodies raised against tyrosine hydroxylase (TH) (rabbit polyclonal, 1:2000; Santa-Cruz) and against DAT (rat monoclonal, 1:100; Merck Millipore). They were maintained for 30 minutes at room temperature and incubated overnight at 4 °C. The primary antibody was removed and slices were rinsed 3 times with chilled PBS. The secondary antibody, Alexa 647 or Alexa 488 (1:100 in PBS/0.5% Triton-X-100/5% Normal Goat Serum), was incubated for 2 h at room temperature, followed by 3 washes in PBS. At least 4 sections from each mouse were mounted with ProLong^®^ Gold Antifade Mountant with DAPI (Thermo Scientific) on SuperFrost glass slides (Thermo Scientific).

### Image collection

Immunohistochemical analysis was performed on n = 3 for each of the experimental groups displayed (WT, KO control, KO treated and KO naïve). Images were taken with a Nikon A1 confocal microscope (Nikon corp, Japan). For the images presented in Fig. 2, a z-stack was acquired and an ‘extended depth of field - EDF’ algorithm^58^ was applied to merge images stacks into final display. ImageJ software^59^ was used; we used EDF algorithm implementation for ImageJ software as previously described^60^.

### Western blot

Protein extraction and preparation of samples for Western blot analysis were performed as described^37^. Briefly, the striatum was dissected from freshly harvested brains. Brain samples were mechanically homogenized in RIPA buffer (50 mmol/l Tris–HCl, pH 7.4/150 mmol/l NaCl/1% Nonidet P-40/0.5% sodium deoxycholate/0.1% SDS; Sigma) plus protease inhibitor mixture (Roche 1873580), and protein concentration was measured using a BCA protein assay kit (Thermo Scientific). Protein extracts (30 μg) were separated by 10% SDS/PAGE and transferred to nitrocellulose membranes. Blots were immunostained overnight at 4 °C with the following primary antibodies: anti-Tyrosine Hydroxylase (Santa Cruz Biotechnology #SC14007, 1:2000 dilution), N-terminal rat anti-DAT (Chemicon #MAB369, 1:500 dilution) and mouse anti-Actin (Sigma-Aldrich #A2853, 1:20000 dilution) and immunostained overnight at 4 °C with primary antibodies. After washing, the membranes were incubated for 2 hours at room temperature with the appropriate secondary antibody (anti-mouse, anti-rabbit or anti-rat). Following secondary antibody incubations, membranes were washed and finally incubated with ECL detection reagent (Amersham RPN2232) for 5 minutes.

### HPLC measurements of the tissue content of monoamines and their metabolites

The protocol for sample preparation for the HPLC determination of tissue monoamines and their metabolites was performed as previously described^19^,^61^. Briefly, whole striatum from WT, KO control and KO treated mice was dissected and frozen in liquid nitrogen. Before tissue content measurement, frozen striatal samples were weighted and homogenized in 40 volumes of 0.1 M HClO_4_. The homogenate was centrifuged at 10 000 g for 10 min and supernatants were filtered through filters (Millipore Ultrafree-MC centrifugal filter units, 0.22 μm). Measurements of DA, 5-HT and metabolites in prepared tissue samples were performed by HPLC paired with electrochemical detection (ALEXYS LC-EC system, Antec Leyden BV, Netherlands) with a 0.7-mm glass carbon electrode (Antec; VT-03). The system was equipped with a reverse-phase column (3-μm particles, ALB-215 C18, 1 × 150 mm, Antec) at a flow rate of 200 μl/min. The mobile phase contained 50 mM H_3_PO_4_, 50 mM citric acid, 8 mM KCl, 0.1 mM EDTA, 400 mg/l octanesulfonic acid sodium salt and 10% (vol/vol) methanol, pH 3.9. The sensitivity of this method permitted the detection of 50 fmol DA. All peaks obtained were normalized to internal standard 3,4-dihydroxybenzylamine (DHBA), and final values for each of the monoamine and metabolites analyzed (DA, Homovanillic Acid – HVA, 3,4-dihydroxyphenylacetic Acid – DOPAC, 5-HT, 5-hydroxyindolacetic acid – 5-HIAA) were expressed as ng/mg wet tissue weight, as reported in Supplementary material (Table S1).

### Fast scan cyclic voltammetry (FSCV) in striatal slices

Briefly, mice were anesthetized with halothane and decapitated. The brains were sectioned in cold carboxygenated artificial cerebrospinal fluid (aCSF) (126 mM NaCl, 2.5 mM KCl, 1.2 mM NaH2PO4, 25 mM NaHCO3, 2.4 mM CaCl2, 11 mM D-glucose, 1.2 mM MgCl2) on a VT1000S vibrating microtome (Leica Microsystems, Nussloch, Germany) at a thickness of 300 μm. Coronal slices containing the dorsal striatum were allowed to recover for at least 1 h at room temperature in carboxygenated aCSF. For recordings, slices were superfused with 32 °C carboxygenated aCSF at a flow rate of 1 ml/min. FSCV recordings started 20 min after transfer to the slice chamber. Carbon fiber electrodes (7 μm diameter, Goodfellow, Huntingdon, England) were made as previously described^62^,^63^. The carbon fibers were trimmed with a scalpel to 80–120 μm under a microscope (Nikon) A carbon fiber microelectrode was inserted into the slice and a twisted bipolar stimulating electrode (Plastics One, Roanoke, VA) was placed on the surface of the brain slice ~200 μm away. The potential of the working electrode was held at −0.4 V and scanned to +1.3 V and back at 300 V/s. Axonal DA release in the striatum was evoked by a single biphasic electrical pulse (1 ms long, 400 μA) every 2 min through a stimulus isolator (AM-system, Carlsborg, WA). Data were filtered to reduce noise. Oxidation and reduction peaks were observed at ~+0.65 V and −0.2 V (vs. Ag/AgCl reference) identifying DA as the released chemical. Electrodes were calibrated in a flow injection system using 1 μM DA (Sigma Aldrich, St. Louis, MO, USA).

### FSCV kinetic analysis

There were several established criteria for choosing which DA signals to use for analysis. Two of these being that there should be no confounding electrical artifacts to interfere with the DA traces, and no pH shifts during recordings, which allow for a flat baseline before stimulation and provide the most uncontaminated DA dynamics as possible, Next, a 10:1 signal to noise ratio was used in order to guarantee the actual signal was separated from background. All of these criteria insured accuracy in analysis. One to two recordings from each experimental group were excluded from analysis based on these criteria.

Data analysis was performed using Demon Voltammetry software described^64^. Briefly, computations were based on user defined positions on current traces for baseline (Pre-Stim cursor), peak (Peak Cursor) and return to baseline (Post-Stim cursor) positions. Half-life values were determined from exponential fit curves based on Peak cursor and Post-Stim cursor positions using a least squares constrained exponential fit algorithm (National Instruments, Milan, Italy)^64^.These measurements were performed on individual traces within each experiment. These numbers were then averaged within each experimental group (WT, KO and KO treated) and reported as mean ± SEM. (n = 4 for WT and KO control; n = 6 for KO treated group). Half-life is considered to be a reliable measure for evaluating changes in striatal DA clearance *in vivo* and *in vitro*. This parameter accurately distinguishes differences in clearance rate similar to other established measures^64^.

### Study approval

All procedures involving animals and their care were carried out in accordance with the guidelines established by the European Community Council (Directive 2010/63/EU of September 22, 2010) and were approved by the Italian Ministry of Health.

### Statistics

Data were obtained from a set of 10 independent stereotaxic surgery events, each having a randomized number of treated and control animals. Western blot and some behavioral assay results were analyzed by analysis of variance (one-way ANOVA) followed by Holm-Sidak *post hoc* analysis. Two-way ANOVA followed by Bonferroni’s *post-*hoc test for multiple comparisons was used to compare groups for specific behavioral comparisons. Mantel-Cox survival analysis was used for survival comparisons. The number of animals used was chosen following the 3R principles (replacement, reduction and refinement) and to ensure statistical power to the analysis. We used GraphPad Prism (version 6) for all analyses, and the null hypothesis was rejected at the 0.05 level of significance.

## Additional Information

**How to cite this article**: Illiano, P. *et al*. Recombinant Adeno-Associated Virus-mediated rescue of function in a mouse model of Dopamine Transporter Deficiency Syndrome. *Sci. Rep.* **7**, 46280; doi: 10.1038/srep46280 (2017).

**Publisher's note:** Springer Nature remains neutral with regard to jurisdictional claims in published maps and institutional affiliations.

## Supplementary Material

Supplementary Information
