# Supplementary material for: Recombinant Adeno-Associated Virus-mediated rescue of function in a mouse model of Dopamine Transporter Deficiency Syndrome
Source: Sci Rep. 2017 Apr 18;7:46280. doi: 10.1038/srep46280 (PMC5394687; doi:10.1038/srep46280)
Supplement: Supplementary Information [file srep46280-s1.pdf]

***Title:* Recombinant Adeno-Associated Virus-mediated rescue of function in a mouse model of Dopamine Transporter Deficiency Syndrome**

Classification: Biological sciences, Neuroscience

Authors: P. Illiano[1], C.E. Bass[2], L. Fichera[3], L. Mus[1], E.A. Budygin[4,5], T.D. Sotnikova[4], D. Leo[1], S. Espinoza[1] and R.R. Gainetdinov[4,6]

Supplementary Materials

***Table S1**. Striatal tissue content*

	Wildtype (n=8)	KO control (n=4)	KO treated (n=5)
Dopamine	8.35 ± 1.13	0.25 ± 0.09	4.91 ± 0.63
HVA	0.61 ± 0.07	2.13 ± 0.75	1.34 ± 0.26
DOPAC	0.72 ± 0.08	0.60 ± 0.20	0.90 ± 0.05
5-HT	0.46 ± 0.04	0.27 ± 0.02	0.43 ± 0.05
5-HIAA	0.24 ± 0.02	0.28 ± 0.04	0.33 ± 0.03

Striatal tissue content data were measured as ratio of area under curve peak detection (expressed in nanograms - ng) over striatal wet tissue weight (expressed in mg). Dopamine and its metabolites Homovanillic Acid (HVA) and 3-4-dihydroxyphenylacetic Acid (DOPAC) were measured, together with serotonin (5-HT) and its metabolite 5-hydroxyindolacetic acid (5-HIAA) for WT animals, KO controls and KO treated mice. All measurements were normalized to DHBA level (internal control of sample preparation quality). Data are expressed mean ± SEM.

Fig. S1. Stereotyped behavior and Western Blot

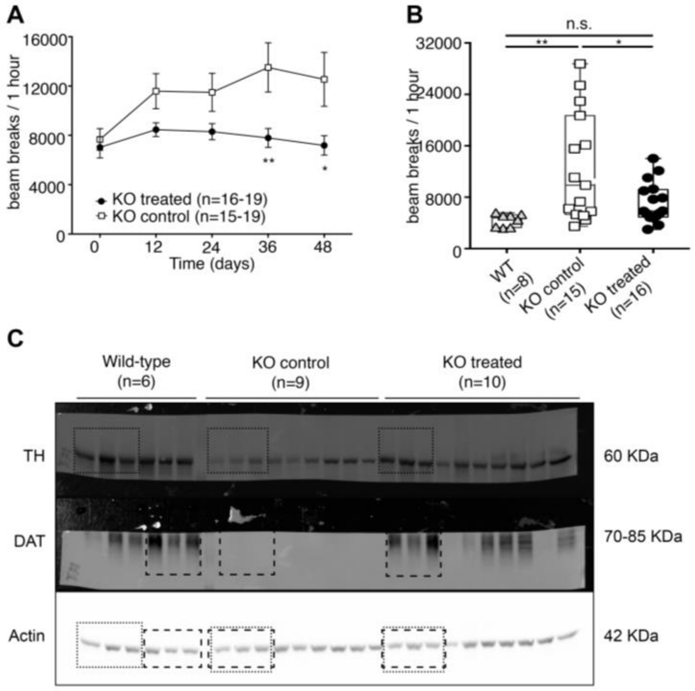

(A) Stereotypies measured in locomotor activity boxes**.** KO control animals (n=15-19) display increased stereotypies starting from Day 36 onwards (Two-way ANOVA, Bonferroni *post-hoc* test, p=0.0069 Day 36; p= 0.0329 Day 48) compared to KO treated mice (n=16-19). **(B)** Reduced stereotyped behaviors of DAT-KO treated mice (48 days after treatment) in comparison to the KO control cohort (Holm-Sidak *post-hoc*, p=0.0249) decreased to levels comparable to those observed in WT littermates (One-way ANOVA, Holm-Sidak *post-hoc* test, p=0.2667). **(C)** Complete Western Blot gels displayed in Fig. 3D. Dashed selections indicate cropped samples shown for Actin, DAT and TH in all of the three groups insofar described. Fig. 3E and 3F display quantitation obtained from all samples shown in these gels. Loading controls were run on the same gel. Data are expressed as the mean ± SEM. One-way ANOVA + Holm-Sidak *post-hoc* and two-way ANOVA + Bonferroni *post-hoc* tests were used for multiple comparisons. **p<0.01; *p<0.05; n.s. – not significant.

__Fig. S2__. Pattern of locomotor activity, gait analysis and clasping behavior

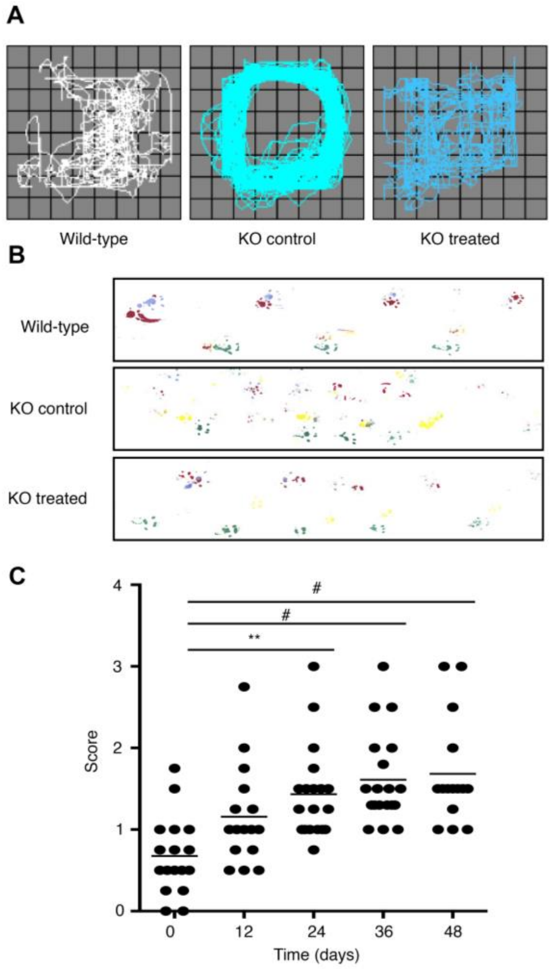

(A) Pattern of locomor activity in locomotor activity boxes. 10 minutes tracking of representative animals from each group (WT, KO control and KO treated) at 36 days after surgery, starting from minute 50:00 to minute 60.00 after placement in the locomotor activity box. **(B)** Footprint analysis. Examples of 30 cm digital scan of footprint for each group at 48 days after rAAV delivery, depicting shorter step and reduced hindpaw distance in KO control mice but not in WT or DAT-KO treated mice. **(C)** Development of clasping behavior in KO control animals, starting from Day 0/pre-surgery and from Day 24 onwards (One-way ANOVA, Holm-Sidak *post-hoc* test $p=0.0012$ Day 24; $p<0.0001$ – Day 36 and Day 48). Data are expressed mean ± SEM. #$p<0.0001$; **$p<0.01$; n.s. – not significant.

Genotyping protocol

Ear punches were collected for genotyping at weaning age ~21 pnd. Briefly, DNA was isolated from biopsies after incubation in 300 µl NaOH 50 mM at 98 °C for 50 minutes. 30 µl of Trizma® hydrocloride 1M (Sigma-Aldrich, T3038) were then added and samples were centrifuged for 6 minutes at 14000 rpm. PCR reaction mix was prepared following Go-Taq protocol (Promega, M3001) using 2 µl of sample extract as DNA template. PCR was performed according to following cycles: 95 °C - 3 minutes, (94 °C - 1 minute, 60 °C 45 seconds, 72 °C – 1 minute) 35 cycles, 72 °C - 5 minutes. PCR primers (DAT-DA: 5'-TGGAGCTCATCTTGGTCAAG -3' ; Neo I: 5'- TTGGCTACCCGTGATATT GC -3'; DA4: 5'- TAC ACCATGCCCTGCACA CA -3') were used to amplify the genomic DNA region containing the NEO cassette as previously reported [5]. PCR products were run on a 2% Agarose gel, and NEO cassette presence in DAT-KO homozygous animals confirmed by visualization of a 800 bp band.
